# New Remedies

**Published:** 1881-10

**Authors:** 


### NEW REMEDIES.

There is always a rage with some of our profession for new medicines. For ourselves, we frankly confess a decided preference for a few very reliable (and old, if necessary) agents rather than for a formidable list of very new and very doubtful ones. The rage above mentioned arises among physicians, from a most laudable motive, that is the advancement of the certainty of therapeutics, and the consequent prompt relief of human suffering, and prolongation of human life. There are those, too, not within our ranks, as honest as ourselves in their endeavor to promote the same objects. None will deny the great assistance druggists and manufacturers have afforded in the furtherance of the objects named. Their confidence in the power of the agents they manipulate is a natural one, but has often led to serious disappointment, when their recommendations have been relied on too implicitly. We fear, too, there are instances where a desire for placing cash in the pocket, has not been subordinate to the placing of new weapons in the hands of the doctor.

We believe it is too much the custom to imagine that some thereapeutical value attaches to every plant that grows, or to every substance that may be bundled into a pill, ground into a powder, or crowded into a handsome extract.

We are willing, we are anxious, that intelligent trial be given any agent which may probably prove a useful remedy. But we must regret and condemn a wholesale and promiscuous laudation of any and all articles before they have been subjected to systematic and extended trials. They need not be advertised merely for the purpose of securing such trial. Not only do such imperfectly tested articles generally lead to disappointment, but they prevent resort to other and older remedies that would probably secure better results. We repeat that a few medicines fully known and well administered will better serve us than a vast army of new-found and transient acquaintances. We fear we are familiar with the names of too many medicines, and with the full power of too few of them.

While we admit that some of our remedies must be empirically used, and were empirically discovered, we insist that intelligent investigation of any remedy must be in the direction of its physiological effects. Nor can its therapeutical value be established except through the intelligent observation of a sufficient series of administrations. This cannot be ascertained by irregular, isolated, or slipshod experiments.

It would be well for every investigator in this field of therapeutics to daily recite "*post hoc, sed non propter hoc.*" Let him remember, too, that without accurate diagnosis, his investigations will afford results worse than useless. Let him remember, further, that a claim of subduing invincible pathological conditions serves to bring himself and remedy into disrepute.

In this connection we may add, that negative results too often follow the administration of a medicine because of the deterioration, inertness, or faulty preparation of the particular specimen used. We are sure the profession is too careless in this respect, and, in consequence, is often outrageously imposed upon.

We should not endorse a remedy until we know it achieved the good results which ensued; nor should we condemn one we have decided to use, until satisfied of its purity and proper preparation, and its fitness for the case in hand. It is best to prescribe remedies that have a definite and positive action, and to endeavor to attain such action before we decide as to the adaptability of the remedy to the condition before us.

To sum up: Be assured your agent is all it purports to be; be secure in your diagnosis, and well fortified in the pathology of the disease; have a definite idea as to what you are to accomplish; see that a sufficient quantity of the article is administered; when the patient has recovered, or you have achieved the desired end, consider well whether your remedy was the means of such achievement; then you may feel authorized to make further investigations, and, after that, report progress.

